# A Note on the Usefulness of Constrained Fourth-Order Latent Differential Equation Models in the Case of Small *T*

**DOI:** 10.1007/s11336-020-09738-x

**Published:** 2020-12-20

**Authors:** Katinka Hardt, Steven M. Boker, Cindy S. Bergeman

**Affiliations:** 1 Department of Psychology, Humboldt-Universität zu Berlin, Unter den Linden 6, 10099 Berlin, Germany; 2 Department of Psychology, University of Virginia, Gilmer Hall Room 102, Charlottesville, VA 22903 USA; 3 Department of Psychology, University of Notre Dame, Corbett Family Hall, Notre Dame, IN 46556 USA

**Keywords:** latent differential equations, damped linear oscillator, continuous time, factor scores, vector field plots

## Abstract

Constrained fourth-order latent differential equation (FOLDE) models have been proposed (e.g., Boker et al. 2020) as alternative to second-order latent differential equation (SOLDE) models to estimate second-order linear differential equation systems such as the damped linear oscillator model. When, however, only a relatively small number of measurement occasions *T* are available (i.e., $T=50$), the recommendation of which model to use is not clear (Boker et al. 2020). Based on a data set, which consists of $T=56$ observations of daily stress for $N=44$ individuals, we illustrate that FOLDE can help to choose an embedding dimension, even in the case of a small *T*. This is of great importance, as parameter estimates depend on the embedding dimension as well as on the latent differential equations model. Consequently, the wavelength as quantity of potential substantive interest may vary considerably. We extend the modeling approaches used in past research by including multiple subjects, by accounting for individual differences in equilibrium, and by including multiple instead of one single observed indicator.

**Electronic supplementary material:**

The online version of this article (10.1007/s11336-020-09738-x) contains supplementary material, which is available to authorized users.

## Introduction

Differential equations (e.g., Boker and Nesselroade 2002; Oud and Singer 2008; Hecht et al. 2019) are of great interest in the study of psychological processes as self-regulating systems. Latent differential equation (LDE) modeling is a technique that allows for estimating the parameters of differential equations models, in which the logically related derivatives of a system are modeled as interrelated latent variables. In the damped linear oscillator (DLO), as an example of a second-order system, the second derivative can be regressed on the zeroth derivative and the first derivative, with the regression coefficients being $\eta$ and $\zeta$, respectively. The parameter $\eta$ is related to the frequency of the oscillation and $\zeta$ to the damping of the oscillation in the system.

**Fig. 1:**
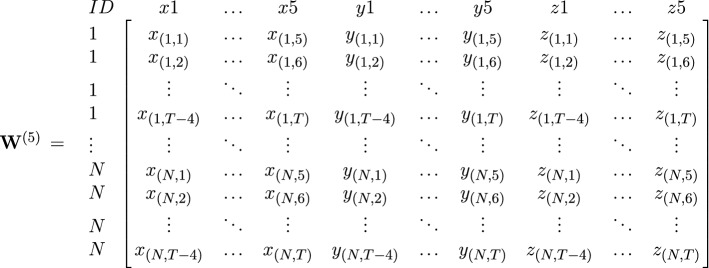
Example for a five-dimensional time delay-embedded data matrix $\mathbf {W}^{(5)}=[\mathbf {X}^{(5)}|\mathbf {Y}^{(5)}|\mathbf {Z}^{(5)}]$ for the three observed time series *X*, *Y*, and *Z*

Constrained fourth-order LDE (FOLDE) models are an alternative to approximating second-order systems. This is possible because FOLDE builds upon three mathematically equivalent sets of second-order equations, in which the second derivative is regressed on the zeroth and first derivatives, the third is regressed on the first and second derivatives, and the fourth is regressed on the second and third derivatives, with each set having regression parameters constrained to $\eta$ and $\zeta$, respectively (for a more detailed derivation of these relationships see Boker et al. 2020, p. 205). Incorporating these higher-order derivatives gives us an extra source of information that has the potential to reduce bias in the parameters of the DLO. Performance of the FOLDE model under simulated, ideal conditions yielded an advantage over the SOLDE model for most cases except when the number of measurement occasions was small (i.e., $T=50$; Boker et al. 2020). In this case, the frequency parameter $\eta$ as well as the likelihood ratio tests benefit from FOLDE, whereas the damping parameter $\zeta$ exhibits larger bias in FOLDE than in SOLDE. Therefore, the recommendation of which model to choose is ‘not so clear’ in this situation. Instead, ‘a safe recommendation is to fit both [SO]LDE and FOLDE to ones data’ (Boker et al. 2020, p. 215).

Before both models can be fit to ones data, the data from a time series need to be time delay embedded in a preprocessing step. For this, the time series is ‘cut’ into snips and restructured in such a way that overlapping samples of the time series are created (see Fig. 1 for an example). How many observations are placed in one row is referred to as the embedding dimension *D*, and the choice of *D* is up to the researchers themselves. Hu et al. (2014) recently found a data-driven way to pick *D*, which will be explained in more detail later.

Based on empirical data, this article showcases that combining these recent developments can add value to the data analysis using LDE. FOLDE here aids in applying Hu et al.’s criterion for identifying an optimal embedding dimension. In addition, this research note demonstrates that carefully setting *D* is important, as substantive conclusions may differ depending on *D*.

## Review of Concepts

### Second-Order Linear Differential Equations

Linear differential equations establish linear relationships between a variable *f* and its derivatives with respect to time (e.g., the first derivative $\dot{f}$ and the second derivative $\ddot{f}$). A second-order linear differential equation model is described by

1 $$\begin{aligned} \ddot{f}_{jt}&=\eta \cdot f_{jt} + \zeta \cdot \dot{f}_{jt}+e_{\ddot{\text {f}}_{jt}} \quad \text {with} \ e_{\ddot{\text {f}}_{jt}}\sim \mathcal {N}({0,V_{e_{\ddot{\text {f}}}}}), \end{aligned}$$

in which index *j* denotes person-specific values, $\eta$ is the *frequency* parameter, which is related to the frequency of the oscillation in a self-regulating system, and $\zeta$ is the *damping* parameter, which governs the amplitude of the oscillation (see Boker 2012; Boker et al. 2016, for a more detailed explanation of the parameter interpretation). The parameters $\eta$ and $\zeta$ do not have person index *j* as they are assumed constant across persons here. Note that all of the variance in LDE latent derivatives is variance that accounts for change over time and does so in the manner of derivatives with respect to time. Therefore, the residual of the latent second derivative, $e_{\ddot{\text {f}}_{jt}}$ having variance $V_{e_{\ddot{\text {f}}}}$, is carried forward in time and contains time-dependent effects not explained by the DLO. For instance, dynamic error can be present or it can be due to model misspecification, that is, when the process we are modeling does not conform to a second-order linear differential equation. In LDE models, however, we cannot distinguish exogenous from endogenous sources, where this residual comes from, as they do not provide us with an explicit estimate of the process-level noise (see e. g., Steele and Ferrer 2011, and Chow et al. 2005, pp. 212–215, for more information on residuals).

### Time Delay Embedding

Before the model parameters can be estimated, the individual time series $X=x_{(1,1)}, x_{(1,2)},\dots , x_{(1,T)},\dots ,x_{(N,1)}, x_{(N,2)},\dots , x_{(N,T)}$ for $i=1,\dots ,T$ measurement occasions ordered within individual $j=1,\dots ,N$ need to be time delay embedded. First, the time series are cut into overlapping segments and then these segments are ‘rowbound.’ For three observed time series *X*, *Y*, and *Z*, Fig. 1 shows that first each of these time series is time delay embedded, and then, the resulting matrices are bound together column-wise. In so doing, a ‘window,’ in which each row comprises a sample of observations of the three time series, builds the basis for estimating the dynamics. This rearrangement of the data increases parameter estimation precision (von Oertzen and Boker 2010) and is relatively robust in sampling interval misspecification (Boker et al. 2018). Whereas the interval between observations (parameter $\tau$) is recommended to be kept at one (Boker et al. 2020), the embedding dimension *D* (and, thus, the ‘width’ of the window) needs to be determined by the researcher. In earlier research (e.g., Boker and Nesselroade 2002), likelihood-based criteria were used to set the embedding dimension. More recently, based on a simulation study, Hu et al. (2014) have suggested that in applied settings, an empirical identification of the optimal *D* should be determined for each model–data combination. The authors recommend plotting the estimated $\eta$ parameter as a function of *D*. Then, the value for *D* that occurs just when the frequency parameter stabilizes is deemed optimal. Or, in simpler words, Hu et al.’s suggestion is to visually identify an ‘elbow’ or ‘reversed elbow’ when plotting $\eta$ by *D*.

**Fig. 2:**
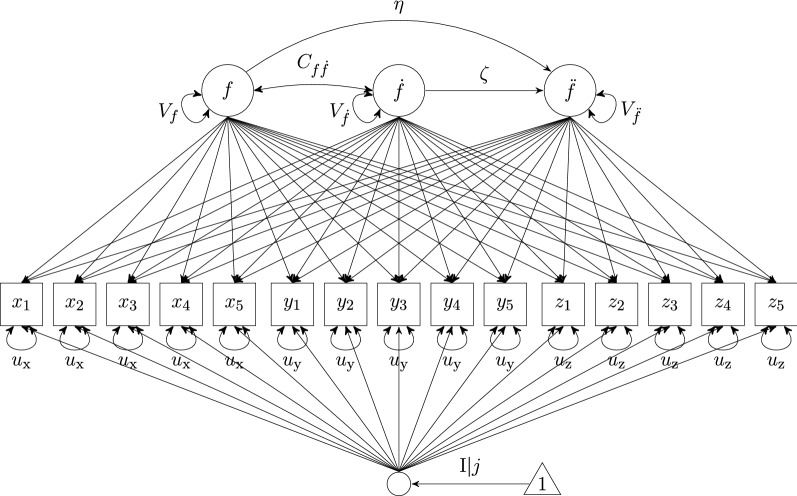
Illustration of multivariate second-order LDE models with individual differences in equilibrium, here based on three observed time series’ *X*, *Y*, and *Z*, each of which was five-dimensionally time delay embedded. Note that the small circle is not an actual latent variable but simply denotes a matrix operation during the estimation

A proper selection of the embedding dimension is crucial. It is only if *D* is chosen appropriately, that Takens’ (1981, pp. 376–379) embedding theorem holds and the dynamics of interest are captured. The lower bound of the embedding dimension is determined by the necessity to identify the model; the upper bound is given by the *Nyquist limit* (e.g., Shannon 1948; Hamming 1998). According to the Nyquist limit, the total time elapsed between the first and the last columns of the time delay-embedded data must not exceed the wavelength of the oscillation in order for the dynamics to be captured (for the specific importance of the Nyquist limit in the context of SOLDE and FOLDE modeling see Boker et al. 2020).

### Model Specification: Multivariate SOLDE and FOLDE Models With Individual Differences in Equilibrium

*SOLDE*.   Latent differential equation models are set up as structural equation models, in which one (in the univariate case) or more (in the multivariate case) observed time series are related to the latent derivative variables. In the multivariate case, we conceive of each observed [time delay-embedded] time series as an indicator of the dynamics of an underlying, latent process. As only the time-structured, common variance of the observed indicators is used, the multivariate model may produce better estimates of the differential equation coefficients. The multivariate SOLDE model with individual differences in equilibrium is depicted in Fig. 2. The zeroth, first, and second derivatives are modeled as latent variables *f*, $\dot{f}$, and $\ddot{f}$, respectively. Each of the manifest variables from the time delay-embedded data matrix loads on each of the latent derivative variables. The loading matrix $\mathbf {L}$ is constrained in a manner so that the common factors are derivatives with respect to time of the rows of the TDE matrix (see Boker 2007, pp. 138–139 for the rationale):

$$\begin{aligned} \mathbf {L} = \begin{bmatrix} 1 &{} -2\Delta t &{} \frac{(-2\Delta t)^2}{2} \\ 1 &{} -1\Delta t &{} \frac{(-1\Delta t)^2}{2} \\ 1 &{} 0 &{} 0 \\ 1 &{} 1\Delta t &{} \frac{(1\Delta t)^2}{2} \\ 1 &{} 2\Delta t &{} \frac{(2\Delta t)^2}{2} \\ l_2 &{} l_2(-2\Delta t) &{} l_2(\frac{(-2\Delta t)^2}{2}) \\ l_2 &{} l_2(-1\Delta t) &{} l_2(\frac{(-1\Delta t)^2}{2}) \\ l_2 &{} 0 &{} 0 \\ l_2 &{} l_2( 1\Delta t) &{} l_2(\frac{(1\Delta t)^2}{2}) \\ l_2 &{} l_2( 2\Delta t) &{} l_2(\frac{(2\Delta t)^2}{2}) \\ l_3 &{} l_3(-2\Delta t) &{} l_3(\frac{(-2\Delta t)^2}{2}) \\ l_3 &{} l_3(-1\Delta t) &{} l_3(\frac{(-1\Delta t)^2}{2}) \\ l_3 &{} 0 &{} 0 \\ l_3 &{} l_3( 1\Delta t) &{} l_3(\frac{(1\Delta t)^2}{2}) \\ l_3 &{} l_3( 2\Delta t) &{} l_3(\frac{(2\Delta t)^2}{2}) \\ \end{bmatrix} \end{aligned}$$

The residual variances of the manifest variables are constrained to be equal for each time series assuming time constant dynamics. Note that as we have multiple observed indicators of the same underlying latent factor, and, thus, multiple observed time series, we model the dynamics of a latent factor *f* instead of each observed variable itself. Frequency and damping of the oscillation are expressed as regression parameters $\eta$ and $\zeta$, respectively, in the structural part of the model (see Fig. 2). Further, the model accounts for individual differences in equilibrium by including a latent intercept (*I*) with mean grouped by individual *j* (Boker et al. 2016). The SOLDE model in Fig. 2 specifies each row belonging to person *j* in the time delay-embedded data matrix as2in which $\mathbf {M}_j$ is the latent intercept mean for person *j*, $\mathbf {K}$ is a matrix of ones, $\mathbf {F}_j$ contains the scores for the derivatives (*f*,  $\dot{f}$, and $\ddot{f}$), and $\mathbf {E}_j$ contains residuals. The residuals in $\mathbf {E}_j$ are unique for each person *j* and normally distributed with mean 0 and variance $u_{\text {TDEvariable}}$. These variances are assumed equal for each embedded indicator in $\mathbf {W}$ belonging to the same time series of either *X*, *Y*, or *Z* in our example (i.e., $u_{\text {X}} \ne u_{\text {Y}} \ne u_{\text {Z}}$). When, as in our example here, the LDE model is applied to a multivariate time series embedded into a TDE matrix, the unique factors may contain a mixture of variance: i) variance that is unrelated to time, and/or ii) variance that is unique to only one of the multivariate time series, and/or iii) variance that is common to all of the multivariate time series but not accounted for by the extracted derivatives. When a FOLDE model is specified rather than a SOLDE model, the higher-order latent derivatives extract more of the reliable variance, and thus, the part of the variance that is not accounted for by the extracted derivatives is reduced.

When multivariate time series are used, the LDE latent derivatives contain common factor variance that exhibits common change over time. One may think of these latent derivatives as common factors with common fate: The variance in their derivatives has a between-row structure in the TDE matrix of multivariate time series. This means that any residual variance in the structural part of the LDE model is also variance with the properties of LDE derivatives, but is not accounted for by the chosen structural model, that is, the linear differential equation that comprises the structural part of the LDE model.

**Fig. 3:**
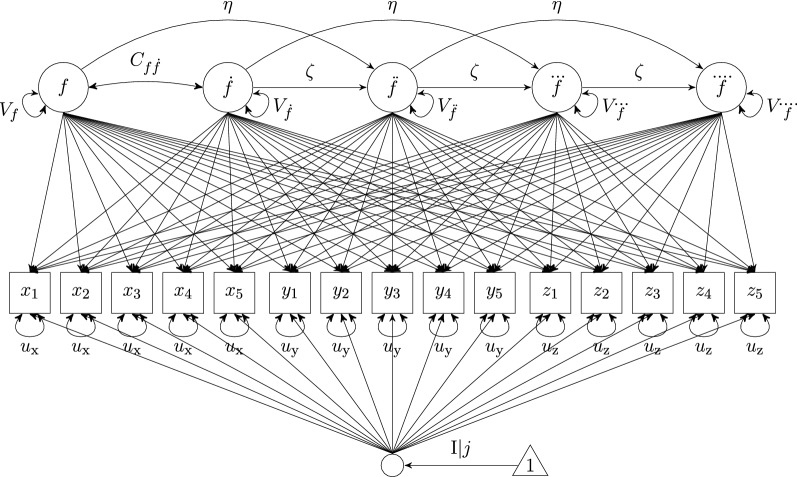
Illustration of multivariate constrained fourth-order LDE models with individual differences in equilibrium, here based on three observed time series’ *X*, *Y*, and *Z*, each of which was five-dimensionally time delay embedded. Note that the small circle is not an actual latent variable but simply denotes a matrix operation during the estimation

*FOLDE*.   The multivariate FOLDE model with individual differences in equilibrium is depicted in Fig. 3 and also formalized by Equation 2, except that $\mathbf {F}_j$ has dimensionality $1\times 5$ and ${\mathbf {L}'}$ has dimensionality $5\times 15$. The corresponding loading matrix is given by

$$\begin{aligned} \mathbf {L} = \begin{bmatrix} 1 &{} -2\Delta t &{} \frac{(-2\Delta t)^2}{2} &{}\frac{(-2\Delta t)^3}{6} &{}\frac{(-2\Delta t)^4}{24} \\ 1 &{} -1\Delta t &{} \frac{(-1\Delta t)^2}{2} &{}\frac{(-1\Delta t)^3}{6} &{}\frac{(-1\Delta t)^4}{24} \\ 1 &{} 0 &{} 0 &{} 0 &{} 0 \\ 1 &{} 1\Delta t &{} \frac{(1\Delta t)^2}{2} &{} \frac{(1\Delta t)^3}{6} &{} \frac{(1\Delta t)^4}{24} \\ 1 &{} 2\Delta t &{} \frac{(2\Delta t)^2}{2} &{} \frac{(2\Delta t)^3}{6} &{} \frac{(2\Delta t)^4}{24} \\ l_2 &{} l_2(-2\Delta t) &{} l_2(\frac{(-2\Delta t)^2}{2}) &{} l_2(\frac{(-2\Delta t)^3}{6}) &{} l_2(\frac{(-2\Delta t)^4}{24} ) \\ l_2 &{} l_2(-1\Delta t) &{} l_2(\frac{(-1\Delta t)^2}{2}) &{} l_2(\frac{(-1\Delta t)^3}{6}) &{} l_2(\frac{(-1\Delta t)^4}{24} ) \\ l_2 &{} 0 &{} 0 &{} 0 &{} 0 \\ l_2 &{} l_2( 1\Delta t) &{} l_2(\frac{(1\Delta t)^2}{2} ) &{} l_2( \frac{(1\Delta t)^3}{6}) &{} l_2( \frac{(1\Delta t)^4}{24}) \\ l_2 &{} l_2( 2\Delta t) &{} l_2(\frac{(2\Delta t)^2}{2} ) &{} l_2( \frac{(2\Delta t)^3}{6}) &{} l_2( \frac{(2\Delta t)^4}{24}) \\ l_3 &{} l_3(-2\Delta t) &{} l_3(\frac{(-2\Delta t)^2}{2}) &{} l_3(\frac{(-2\Delta t)^3}{6}) &{} l_3(\frac{(-2\Delta t)^4}{24} ) \\ l_3 &{} l_3(-1\Delta t) &{} l_3(\frac{(-1\Delta t)^2}{2}) &{} l_3(\frac{(-1\Delta t)^3}{6}) &{} l_3(\frac{(-1\Delta t)^4}{24} ) \\ l_3 &{} 0 &{} 0 &{} 0 &{} 0 \\ l_3 &{} l_3( 1\Delta t) &{} l_3(\frac{(1\Delta t)^2}{2} ) &{} l_3( \frac{(1\Delta t)^3}{6}) &{} l_3( \frac{(1\Delta t)^4}{24}) \\ l_3 &{} l_3( 2\Delta t) &{} l_3(\frac{(2\Delta t)^2}{2} ) &{} l_3( \frac{(2\Delta t)^3}{6}) &{} l_3( \frac{(2\Delta t)^4}{24}) \\ \end{bmatrix} \end{aligned}$$

## SOLDE and FOLDE Modeling Applied to Data With Small *T*

### Data

The data for the substantive example come from a subsample of $N=44$ individuals aged between 65 and 79 years ($M=70.23, \ \text {SD}=3.44$) who participated in the *Notre Dame Study of Health and Well-being* (Bergeman and Deboeck 2014). The 56-day daily diary study included the Perceived Stress Scale (PSS, 10 items; Cohen and Williamson 1988) measuring how stressful participants experienced daily life. Previous studies have used data from the same study to analyze dynamics therein by means of damped linear oscillator models (e.g., Montpetit et al. 2010; Deboeck and Bergeman 2013; Bergeman and Deboeck 2014; Deboeck 2020). Further, in order to ensure stable parameter estimates, three individuals were excluded whose responses were extremely unlikely for a second-order DLO model. Thus, data from $N=41$ individuals entered the analyses.

### Analyses

To demonstrate the merit and consequences of applying FOLDE modeling to data with small *T*, multivariate SOLDE and FOLDE models allowing for individual differences in the perceived stress equilibrium were fit to the data. We also analyzed univariate models based on one single PSS composite (sum score) to cross-validate our results and to align with previous research using data from the same study. The results mostly revealed the same patterns (see Online Resource A), differences will be reported briefly. Figures 2 and 3 depict diagrams of the models except that in our case 10 single indicators were available instead of three.^1^ The embedding dimensions were set to $D=[5..9]$.^2^ All analyses were conducted in the software environment R (R Core Team 2018, version 3.5.2), and LDE models were fit using the R package OpenMx (Neale et al. 2016, Neale, et al. 2018, version 2.12.2). Assuming that missing data were missing at random, we employ full maximum likelihood estimation to handle missing data. We relied on OpenMx default values for model convergence, but used the function mxTryHard() with 30 extra attempts to reach model convergence. In the extra attempts, parameter estimates from the previous attempt were perturbed by random draws from a uniform distribution and then used as starting values for the next attempt. Code for fitting multivariate SOLDE and FOLDE models with individual differences in equilibrium is provided in Online Resource C.

The following outcomes are considered: (1) the identification of a reversed elbow in the plotted $\eta$ estimates according to Hu et al., and, thus, the stabilization of $\eta$; (2) the $\zeta$ estimates; if $\eta$ and $\zeta$ are unstable across embedding dimensions, so will be the estimated wavelength of the oscillation as a function of $\eta$ and $\zeta$; and (3) the global fit of SOLDE and FOLDE models by means of likelihood ratio tests with two degrees of freedom (see Boker et al. 2020, p. 6).

### Results

**Fig. 4:**
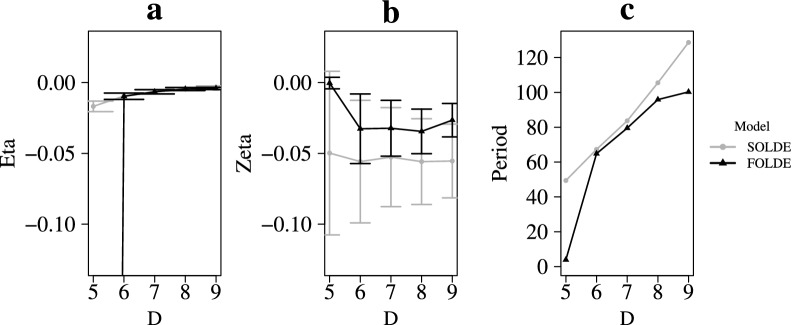
Results for multivariate SOLDE and FOLDE modeling of stress regulation for the three outcome criteria by embedding dimension; *D* $=$ embedding dimension. (a) Frequency ($\eta$), point estimates $\pm \ SE$. Note that $\eta$ $=$ $-2.58$ for FOLDE at *D* $=$ 5. (b) Damping ($\zeta$), point estimates $\pm \ SE$. (c) Period

Results generally indicate an advantage of applying FOLDE when it comes to determining the optimal embedding dimension according to Hu et al. (2014). Figure 4a shows that the frequency estimates $\eta$ do not exhibit a reversed elbow in SOLDE modeling, but we can clearly identify such for the FOLDE modeling at $D=6$. If we only applied SOLDE models, we would not know from which embedding dimension we should interpret our model results. The damping parameter $\zeta$ has only small variability from $D=6$ onward in both SOLDE and FOLDE models (Fig. 4b). Yet, the absolute value of $\zeta$ is different with the SOLDE model, indicating stronger damping in the self-regulation process. Further, at $D=6$, parameter estimates for the two LDE models are almost identical. Substantively, we come to a similar conclusion with regard to the estimated wavelength of the oscillating stress regulation at this embedding dimension, that is, 67.275 days according to SOLDE and 64.833 days according to FOLDE (Fig. 4c). There is considerable variance in the estimated wavelength, however, depending on the embedding dimension. For SOLDE, it ranges from 49.418 to 128.683 days, and for FOLDE, it ranges from 3.911 to 111.545 days. We could arrive at substantively very different conclusions if we chose the embedding dimension arbitrarily. Model comparisons based on $\chi _{\text {diff}}^2\;$- tests prefer the FOLDE over the SOLDE model from $D=6$ onward.

## Discussion

This research note illustrates the need of carefully picking the embedding dimension in LDE modeling and the potential aid of FOLDE therein. When applying the data-driven method by Hu et al. (2014) to determine the optimal embedding dimension, SOLDE left us uncertain about which *D* to choose, whereas FOLDE yielded an optimal *D*. While extending the FOLDE model used by Boker et al. to include multiple observed indicators and multiple subjects, we complemented previous, simulation-based research on FOLDE performance under ideal conditions (Boker et al. 2020) with a perspective from a practical point of view under imperfect conditions using empirical data. Unlike most of the previous methodological studies that build upon time delay embedding, we not only studied effects of *D* on the frequency and damping parameters, but we also specifically inspected the wavelength, which is a function of those two parameters, as a substantively meaningful quantity. In the way frequency and damping parameters are combined, seemingly small differences in each of the two parameters already lead to considerable differences in the wavelength. Consequently, substantive conclusions and implications based on the wavelength may challenge theory if *D* has not been chosen reasonably. For example, in the study of the female menstrual cycle (e.g., Klump et al. 2013; Boker et al. 2014), there is a strong, biologically rooted theory about the length of the cycle. If we then find the wavelength to be varying across *D*, the results may even be contradictory to biological theory. Our results indicate that fitting FOLDE in addition to SOLDE models can aid in checking for parameter stabilization and identifying an embedding dimension deemed optimal for a given model–data combination.

Several issues and limitations should be discussed. To begin with, one issue not mentioned thus far is run time. Whereas run time did not take longer than 2 min in the univariate daily stress modeling (see Online Resource A), it noticeably increased in the multivariate modeling (ranging from 16 min to 1.8 h for SOLDE and from 15 min to 2 h for FOLDE). As the latter are quite complex and difficult to estimate, multiple fitting attempts may be necessary to reach convergence. For this reason, total run time for one multivariate FOLDE model may easily exceed several hours or even days.

Although an applied data analysis has the appeal of being easily comprehensible and therefore serves our illustrative purpose well, it does not allow for studying general mechanisms under controlled conditions as comprehensive simulations do. For instance, we do not know how increased model complexity in the multivariate case, the number of indicators, absolute parameter values, LDE model, and the time delay embedding interplay. Neither do we know how sensitive or robust the chosen modeling approaches in the given situations are to slight model misspecification (e. g., some individuals whose dynamics follow slightly different model parameters).

Another issue concerns the small *T* situation in the presence of multiple subjects. On the one hand, multiple subjects can be an additional source of information for model estimation and can compensate for small *T* to some degree (Hecht and Zitzmann 2020). On the other hand, boundary effects may occur (i.e., the first and the last couple of rows in a fully embedded time series may exhibit bias that does not cancel, especially with short time series and large embedding dimensions; Boker et al. 2018) and add up. It is important to investigate how these two factors act or interact in LDE modeling.

Another desideratum not discussed thus far is the linkage of our group-level results to the individual level. For instance, this can be accomplished by means of vector field plots, which rely on factor scores (see Deboeck 2020 for an example using methods related to LDE). In LDE models, just as in any other structural equation model, factor scores can be obtained subsequently after having estimated the model parameters; for example, the regression or Bartlett methods are readily available in nearly every standard statistical software package. Details on factor score generation as well as vector field plots for a few example cases from our data are provided in Online Resource D. The general result across LDE model, model type (univariate versus multivariate), and factor score method is that vector field plots based on the regression method more clearly reveal dynamics than the Bartlett-based vector field plots. This is a plausible finding given that the regression method accounts for the covariance matrix of the latent derivative variables, which is at the core of LDE modeling, whereas the Bartlett method does not. Using empirical data, however, we can only inspect the appearance and interpretability of vector field plots based on factor scores. In order to also assess their finite sample properties, simulations would be required.

## Electronic supplementary material

Below is the link to the electronic supplementary material.

Supplementary material 1 (pdf 179 KB)

Supplementary material 2 (pdf 145 KB)

Supplementary material 3 (r 22 KB)

Supplementary material 4 (r 3 KB)

Supplementary material 5 (r 13 KB)

Supplementary material 6 (pdf 363 KB)

## Footnotes

1 As the multivariate FOLDE model with interindividal differences in equilibrium has never been under study in the literature to the best of our knowledge, we conducted a small proof-of-concept simulation to ensure the basic functioning of this model (see Online Resource B).

2 In our case, this range of *D* is sufficient to identify a ‘reversed elbow.’
