# Supplementary material for: A Note on the Usefulness of Constrained Fourth-Order Latent Differential Equation Models in the Case of Small T
Source: Psychometrika. 2020 Dec 20;85(4):1016–27. doi: 10.1007/s11336-020-09738-x (PMC7826318; doi:10.1007/s11336-020-09738-x)
Supplement: Supplementary file 1 — Supplementary material 1 (pdf 179 KB) [file 11336_2020_9738_MOESM1_ESM.pdf]

Appendix

Online Resource A: Method and Results for Univariate PSS Modeling

In this Online Resource, we report method and results for the univariate PSS modeling. This Online Resource serves to cross-validate our results found for the multivariate modeling and also to align with previous research using data from the same study.

Review of Concepts Based on the Univariate LDE Modeling

In order to convey a general understanding of the data that are being modeled with SOLDE and FOLDE models with individual differences in equilibrium, Figure A1 displays the raw scores of the 10 PSS indicators, the individual mean score as well as the estimated equilibrium value for one example case from our data. The fluctuations of the scores around the equilibrium as well as the fading out thereof can clearly be seen. Substantively, that person's stress regulation obviously conforms to a damped linear oscillator.

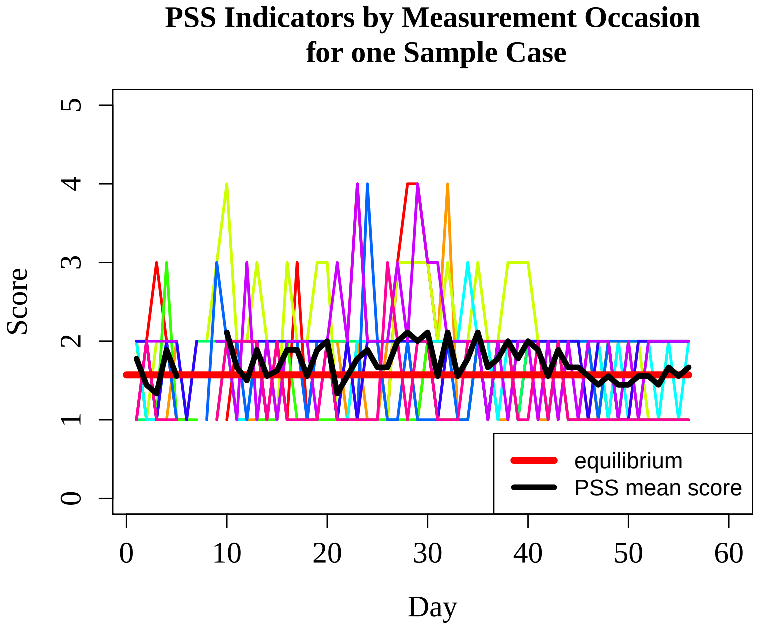

Figure A1. Time series plots of the 10 PSS indicators (thin rainbow-coloured lines), the PSS mean score (thick black line) and the individual equilibrium estimated in the multivariate SOLDE model (thick red line).

Time-Delay Embedding

To give an example, suppose a time series of one variable, such as a composite for perceived stress, is given by $X = x_{(1,1)}, x_{(1,2)}, \ldots, x_{(1,T)}, \ldots, x_{(N,1)}, x_{(N,2)}, \ \ldots, x_{(N,T)}$ for $i = 1, \ldots, T$ measurement occasions ordered within individual $j = 1, \ldots, N$; then, the 5-dimensional time-delay embedded matrix $\mathbf{X}^{(5)}$ is given by:

$$\mathbf{X}^{(5)} = \begin{array}{c} ID \\ 1 \\ 1 \\ 1 \\ 1 \\ \vdots \\ N \\ N \\ N \end{array} \begin{array}{c} \begin{array}{ccccc} x1 & x2 & x3 & x4 & x5 \end{array} \\ \left[\begin{array}{ccccc} x_{(1,1)} & x_{(1,2)} & x_{(1,3)} & x_{(1,4)} & x_{(1,5)} \\ x_{(1,2)} & x_{(1,3)} & x_{(1,4)} & x_{(1,5)} & x_{(1,6)} \\ \vdots & \vdots & \vdots & \vdots & \vdots \\ x_{(1,T-4)} & x_{(1,T-3)} & x_{(1,T-2)} & x_{(1,T-1)} & x_{(1,T)} \\ \vdots & \vdots & \vdots & \vdots & \vdots \\ x_{(N,1)} & x_{(N,2)} & x_{(N,3)} & x_{(N,4)} & x_{(N,5)} \\ \vdots & \vdots & \vdots & \vdots & \vdots \\ x_{(N,T-4)} & x_{(N,T-3)} & x_{(N,T-2)} & x_{(N,T-1)} & x_{(N,T)} \end{array} \right] \end{array}$$

Model Specification

In univariate SOLDEs, the zeroth, first and second derivative are modeled as latent variables f, $\dot{f}$ and $\ddot{f}$, respectively. Each of the manifest variables from the time-delay embedded data matrix loads on each of the latent derivative variables. The loading matrix $\mathbf{L}$ is fixed in such a way that latent derivatives are estimated (see Boker, 2007, pp.138–139 for the rationale):

$$\mathbf{L} = \begin{bmatrix} 1 & -2\Delta t & \frac{(-2\Delta t)^2}{2} \\ 1 & -1\Delta t & \frac{(-1\Delta t)^2}{2} \\ 1 & 0 & 0 \\ 1 & 1\Delta t & \frac{(1\Delta t)^2}{2} \\ 1 & 2\Delta t & \frac{(2\Delta t)^2}{2} \end{bmatrix}$$

The error variances of the manifest variables are constrained to be equal for each time series assuming time-constant dynamics. Frequency and damping of the oscillation are expressed as regression parameters η and ζ, respectively, in the structural part of the model (see Figure A2). As in the multivariate LDE modeling, individual differences in equilibrium are accounted for by including a latent intercept (I) with mean grouped by individual (j) (Boker, Staples, & Hu, 2016).mThe SOLDE model in Figure A2 specifies each row belonging to person j in the time-delay embedded data matrix as

$$\underset{1\times 5}{\mathbf{X}_j} = \underset{1\times 1}{\mathbf{M}_j} \cdot \underset{1\times 5}{\mathbf{K}} + \underset{1\times 3}{\mathbf{F}_j} \cdot \underset{3\times 5}{\mathbf{L}'} + \underset{1\times 5}{\mathbf{E}_j} \tag{1}$$

where $\mathbf{M}_j$ is the latent intercept mean for person j, $\mathbf{K}$ is a matrix of ones, $\mathbf{F}_j$ contains the scores for the derivatives (f, $\dot{f}$ and $\ddot{f}$), $\mathbf{E}_j$ contains residuals e_{x_j}, which are unique for each person j and for each of the embedding variables $x_1, \ldots, x_5$ but have equal variance u_{x}, and all other terms as defined before.

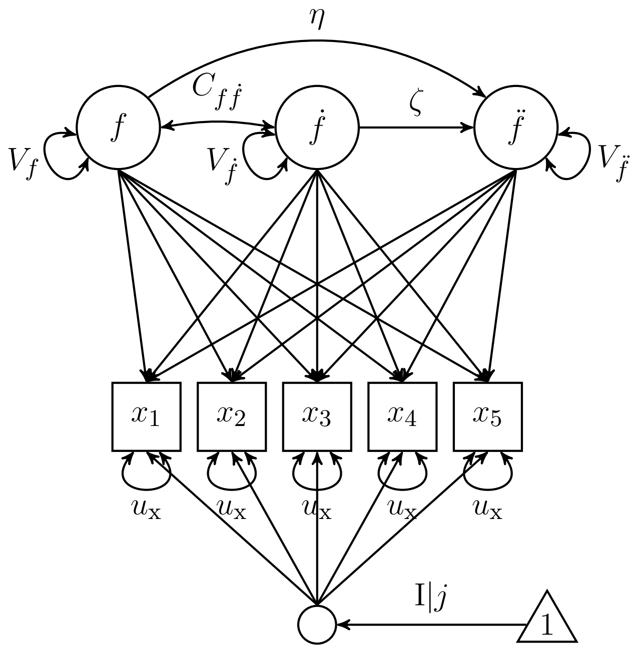

Figure A2. Univariate Second Order LDE with Individual Differences in Equilibrium.

Univariate FOLDE models additionally contain the third and the fourth derivative as latent variables $\dddot{f}$ and $\ddddot{f}$, respectively. Accordingly, the loading matrix is altered to:

$$\mathbf{L} = \begin{bmatrix} 1 & -2\Delta t & \frac{(-2\Delta t)^2}{2} & \frac{(-2\Delta t)^3}{6} & \frac{(-2\Delta t)^4}{24} \\ 1 & -1\Delta t & \frac{(-1\Delta t)^2}{2} & \frac{(-1\Delta t)^3}{6} & \frac{(-1\Delta t)^4}{24} \\ 1 & 0 & 0 & 0 & 0 \\ 1 & 1\Delta t & \frac{(1\Delta t)^2}{2} & \frac{(1\Delta t)^3}{6} & \frac{(1\Delta t)^4}{24} \\ 1 & 2\Delta t & \frac{(2\Delta t)^2}{2} & \frac{(2\Delta t)^3}{6} & \frac{(2\Delta t)^4}{24} \end{bmatrix}$$

Figure A3 depicts the univariate FOLDE model with individual differences in equilibrium. The model is also given by Equation 1 except that $\mathbf{F}_j$ now has dimensionality 1×5 and $\mathbf{L}'$ has dimensionality 5×5.

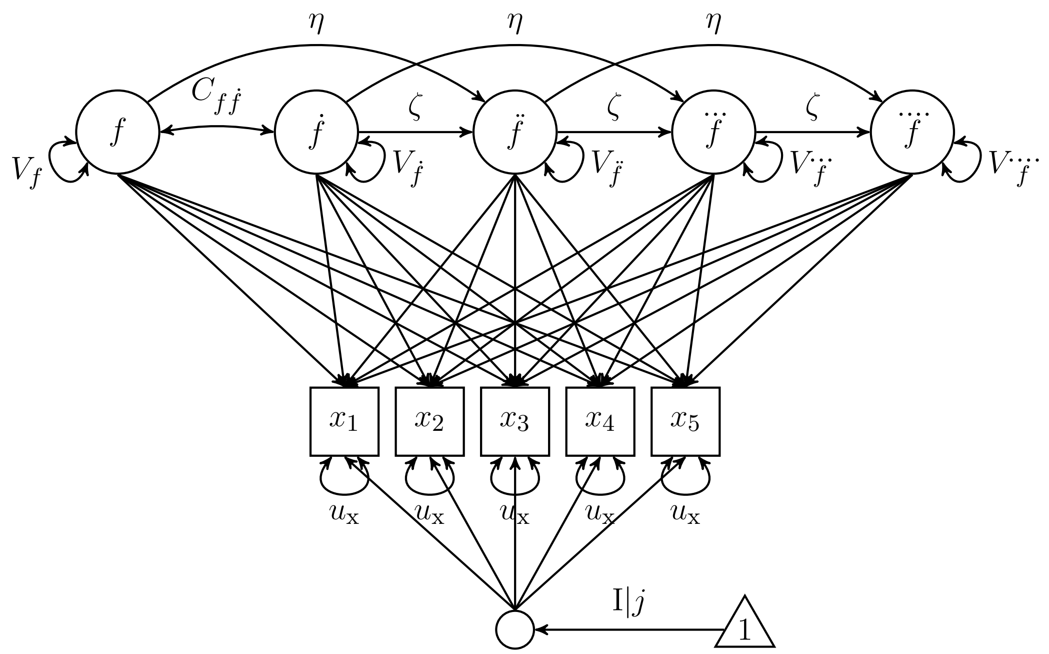

Figure A3. Univariate Constrained Fourth Order LDE with Individual Differences in Equilibrium.

Analyses

The embedding dimensions for the univariate SOLDE and FOLDE models were set to $D = [5..15]$.

Results

Figure A4 shows the results for the estimated parameters η, ζ, and the wavelength as a function of the embedding dimension in univariate SOLDE and FOLDE modeling. Whereas the frequency parameter η appears stable across all embedding dimensions in SOLDE modeling, we can clearly identify a reversed elbow for the FOLDE modeling at $D = 10$. If we only applied the SOLDE models, we would not know based on which embedding dimension we should interpret our model results. Further, as the damping parameter varies widely across D in SOLDE modeling, we would also arrive at very different conclusions regarding the wavelength of the period. Deciding for $D = 10$, parameter estimates for SOLDE and FOLDE models are close to each other and we would come to similar substantive conclusions. Model comparisons based on χ^2_{diff} - tests prefer the FOLDE over the SOLDE model from $D = 10$ onward.

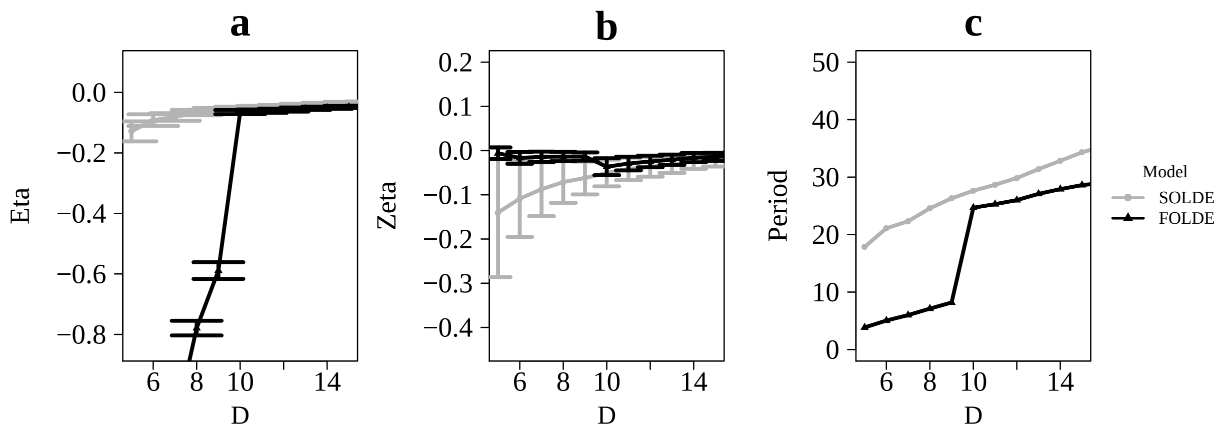

Figure A4. Results for multivariate SOLDE and FOLDE modeling of stress regulation for the three outcome criteria by embedding dimension; D = embedding dimension. (a) Frequency (η) point estimates ± SE. (b) Damping (ζ) point estimates ± SE. (c) Period..

Run Time

Run time is generally not an issue in univariate daily stress modeling (ranging from 4secs to 1.5mins for SOLDE and from 6secs to 2mins for FOLDE on a modern laptop).

References

Boker, S. M. (2007). Specifying latent differential equations models. In S. M. Boker & M. J. Wenger (Eds.), *Data analytic techniques for dynamical systems* (pp. 131–159). Mahwah, NJ: Lawrence Erlbaum Associate Publishers.

Boker, S. M., Staples, A. D., & Hu, Y. (2016). Dynamics of change and change in dynamics. *Journal for Person-Oriented Research*, *2*(1–2), 34–55. doi:10.17505/jpor.2016.05
