# Supplementary material for: A Note on the Usefulness of Constrained Fourth-Order Latent Differential Equation Models in the Case of Small T
Source: Psychometrika. 2020 Dec 20;85(4):1016–27. doi: 10.1007/s11336-020-09738-x (PMC7826318; doi:10.1007/s11336-020-09738-x)
Supplement: Supplementary file 2 — Supplementary material 2 (pdf 145 KB) [file 11336_2020_9738_MOESM2_ESM.pdf]

Appendix

Online Resource B: Proof of Concept Simulation for the Multivariate SOLDE and FOLDE Models With Individual Differences in Equilibrium

Purpose and Previous Research

To the best of our knowledge, the multivariate FOLDE model with individual differences in equilibrium that we used has never been studied and reported elsewhere before. However, model functioning and parameter quality were shown before in the following studies:

- univariate FOLDE modeling without individidual differences in equilibrium (Boker, Moulder, & Sjobeck, 2020; Boker, 2007)
- multivariate FOLDE modeling without individual differences in equilibrium applied to an empirical data set (Chow, Ram, Boker, Fujita, & Clore, 2005)
- multivariate SOLDE modeling without individual differences in equilibrium (von Oertzen & Boker, 2010; Boker, Neale, & Rausch, 2004; Boker, Tiberio, & Moulder, 2018)
- univariate SOLDE modeling with individual differences in equilibrium (Boker, Staples, & Hu, 2016)

In order to ensure that our specific model also yields reliable parameter estimates, we conducted a small proof of concept simulation that provides evidence for the general functioning of the multivariate SOLDE and FOLDE models with individual differences in equilibrium. For this purpose, the DLO parameters from our empirical data analysis were used as data generating input parameters.

Methods

Data were generated for $N = 41$ persons measured on 10 observed variables for $T = 56$ occasions conforming to a DLO with $\eta = -0.01$ and $\zeta = -0.03$. The loadings varied between 0.6 and 1. The time-delay embedding dimension was 6, the tau and deltaT parameters were 1.

The simulation was conducted in the software environment R (R Core Team, 2019, version 3.6.2) using the package deSolve (Soetaert, Petzoldt, & Setzer, 2010, version 1.28) to generate data conforming to a DLO and using the package OpenMx (Neale et al., 2016; Boker et al., 2019, version 2.17.2) and the NPSOL optimizer to estimate the LDE models

under multiple core usage. The simulation was run on a standard laptop and the procedure was replicated 400 times. Since multivariate LDE models are complex and hard to fit, we employed the `mxTryHard()` function with 5 extra attempts to reach model convergence. For model convergence, we relied on OpenMx default criteria. The full simulation code is provided as part of Online Resource A. Run time for this small simulation was already approximately 38.5h on a standard laptop.

Model performance is evaluated with respect to convergence, bias, variance, and root mean square error (RMSE). As measure of bias, the relative bias is reported and as measure of the variance, the standard deviation of an estimate across replications is reported.

Results

Table B1 displays the results of this proof of concept simulation. As can be seen, model performance is in general good for the simulated scenarios for both the SOLDE and the FOLDE model. The biases are small and well below ±10%, which is still considered to be acceptable by some researchers (Muthen & Muthen, 2002).

In contrast to Boker et al. (2020), who relied on univariate models for one single subject, we found that for the multivariate, multisubject SOLDE approach to modeling the DLO, convergence was slightly higher, bias for the frequency parameter was slightly smaller and variances for frequency and damping parameters were also smaller than for the FOLDE approach. The RMSE combining bias and variance is also slightly smaller for SOLDE than for FOLDE with respect to the frequency parameter and almost equal for SOLDE and FOLDE with respect to the damping parameter. However, in general, differences are rather small and both models work very well in the given context. In comparison, Boker et al. (2020) report relative biases of the frequency and damping parameter for the SOLDE approach between 7 and 18% across all conditions and for the FOLDE approach between 0.1 and 11%. The differences may be due to the increased model complexity as a consequence of the multivariate measurement of the latent construct. However, the interplay between multivariate LDE model, model complexity (e.g., in terms of number of indicators) and DLO parameter values can only be investigated using fully-blown Monte Carlo simulation studies.

In conclusion, for the our purpose, our proof of concept simulation has ensured that the SOLDE and FOLDE models work well in the simulated context.

Table B1
Simulation Results

Outcome	SOLDE	FOLDE
Convergence (%)	96.25	84
relative bias$_\eta$ (%)	0.82642	1.89747
relative bias$_\zeta$ (%)	2.63169	2.12508
SD_η	0.00045	0.08721
SD_ζ	0.00246	0.00258
$RMSE_\eta$	0.00046	0.08722
$RMSE_\zeta$	0.00265	0.00276

References

Boker, S. M. (2007). Specifying latent differential equations models. In S. M. Boker & M. J. Wenger (Eds.), *Data analytic techniques for dynamical systems* (pp. 131–159). Mahwah, NJ: Lawrence Erlbaum Associate Publishers.

Boker, S. M., Moulder, R. G., & Sjobeck, G. R. (2020). Constrained fourth order latent differential equation reduces parameter estimation bias for damped linear oscillator models. *Structural Equation Modeling: A Multidisciplinary Journal*, *27*(2), 202–218. doi:10.1080/10705511.2019.1641816

Boker, S. M., Neale, M., & Rausch, J. (2004). Latent differential equation modeling with multivariate multi-occasion indicators. In R. Lowen, K. van Montfort, J. Oud, & A. Satorra (Eds.), *Recent Developments on Structural Equation Models* (Vol. 19, pp. 151–174). Dordrecht: Springer Netherlands. doi:10.1007/978-1-4020-1958-6_9

Boker, S. M., Neale, M. C., Maes, H. H., Wilde, M. J., Spiegel, M., Brick, T. R., ... The Regents of the University of California. (2019). Openmx version 2.17.2 [Computer software manual].

Boker, S. M., Staples, A. D., & Hu, Y. (2016). Dynamics of change and change in dynamics. *Journal for Person-Oriented Research*, *2*(1–2), 34–55. doi:10.17505/jpor.2016.05

Boker, S. M., Tiberio, S. S., & Moulder, R. G. (2018). Robustness of time delay embedding to sampling interval misspecification. In K. v. Montfort, J. H. L. Oud, & M. C. Voelkle (Eds.), *Continuous time modeling in the behavioral and related sciences.* New York: Springer.

Chow, S.-M., Ram, N., Boker, S. M., Fujita, F., & Clore, G. (2005). Emotion as a thermostat: Representing emotion regulation using a damped oscillator model. *Emotion*, *5*(2), 208–225. doi:10.1037/1528-3542.5.2.208

Muthen, L. K., & Muthen, B. O. (2002). How to use a Monte Carlo study to decide on sample size and determine power. *Structural Equation Modeling: A Multidisciplinary Journal*, *9*(4), 599–620. doi:10.1207/S15328007SEM0904_8

Neale, M. C., Hunter, M. D., Pritikin, J. N., Zahery, M., Brick, T. R., Kirkpatrick, R. M., . . . Boker, S. M. (2016). OpenMx 2.0: Extended Structural Equation and Statistical Modeling. *Psychometrika*, *81*(2), 535–549. doi:10.1007/s11336-014-9435-8

R Core Team. (2019). R: A language and environment for statistical computing, r version 3.6.2 [Computer software manual]. Vienna, Austria. Retrieved from `https://www.R-project.org/`

Soetaert, K., Petzoldt, T., & Setzer, R. W. (2010). Solving differential equations in R: Package deSolve. *Journal of Statistical Software*, *33*(9), 1–25. Retrieved from `http://www.jstatsoft.org/v33/i09` doi:10.18637/jss.v033.i09

von Oertzen, T., & Boker, S. M. (2010). Time delay embedding increases estimation precision of models of intraindividual variability. *Psychometrika*, *75*(1), 158–175. doi:10.1007/s11336-009-9137-9
