# Supplementary material for: A Note on the Usefulness of Constrained Fourth-Order Latent Differential Equation Models in the Case of Small T
Source: Psychometrika. 2020 Dec 20;85(4):1016–27. doi: 10.1007/s11336-020-09738-x (PMC7826318; doi:10.1007/s11336-020-09738-x)
Supplement: Supplementary file 6 — Supplementary material 6 (pdf 363 KB) [file 11336_2020_9738_MOESM6_ESM.pdf]

Appendix

Online Resource D: Using Vector Field Plots Based on Individual Scores to Illustrate Individual Dynamics

Tracing Individual Dynamics Based on Individual Scores

Individual Dynamics can be traced by using individual scores, which are individual values for the latent derivative variables. Common methods to obtain individual scores are the *Bartlett* (Bartlett, 1937) and the *regression* (Thomson, 1938; Thurstone, 1934) methods. Individual scores according to the *Bartlett method* are given by

$$\hat{\mathbf{f}}_{\mathrm{B}_j} = (\mathbf{L}' \cdot \mathbf{U}^{-1} \cdot \mathbf{L})^{-1} \cdot \mathbf{L}' \cdot \mathbf{U}^{-1} \cdot (\mathbf{W}_j^{(D)} - \mathbf{M}_j)' \ , \tag{1}$$

where all terms are as defined before. By subtracting $\mathbf{M}_j$, the scores are in deviation form, that is, deviation from the individual equilibrium.
According to the *regression method*, individual scores are given by

$$\hat{\mathbf{f}}_{\mathrm{R}_j} = \mathrm{cov}(\mathbf{F}) \cdot \mathbf{L}' \cdot \mathcal{E}(\mathrm{cov}(\mathbf{W}_j^{(D)}))^{-1} \cdot (\mathbf{W}_j^{(D)} - \mathbf{M}_j)' \ , \tag{2}$$

where $\mathrm{cov}(\mathbf{F})$ is the implied covariance matrix of the latent derivatives, $\mathcal{E}(\mathrm{cov}(\mathbf{W}_j^{(D)}))$ is the expected covariance matrix of the data given by $\mathcal{E}(\mathrm{cov}(\mathbf{W}_j^{(D)})) = \mathbf{L} \cdot \mathrm{cov}(\mathbf{F}) \cdot \mathbf{L}' + \mathbf{U}$, where $\mathbf{U}$ contains all the variances u of the embedding variables. Note that in general, the result of computing individual scores in this context is a matrix $\hat{\mathbf{f}}_j$ containing estimated realizations for the latent derivatives (zeroth, first and second for SOLDE and zeroth, first, second, third and fourth for FOLDE) for each row in the time-delay embedded data matrix for every person j.

Results on the Individual Dynamics for the Example Case Based on the Multivariate LDE Modeling

When visualizing dynamics at the individual level using vector field plots, appearance and interpretability of the patterns depend on LDE model and individual score method (see Figure D1 for one example case and Online Resource C for more). We learn that the stress regulation system of our example is spiraling down towards a point near a PSS factor score of 0.35 and that it takes much to perturb this rather stable system. Whereas the regression method based scores yield recognizable and almost identical patterns of dynamics, the Bartlett method based scores convey a less readily apparent picture of individual dynam-

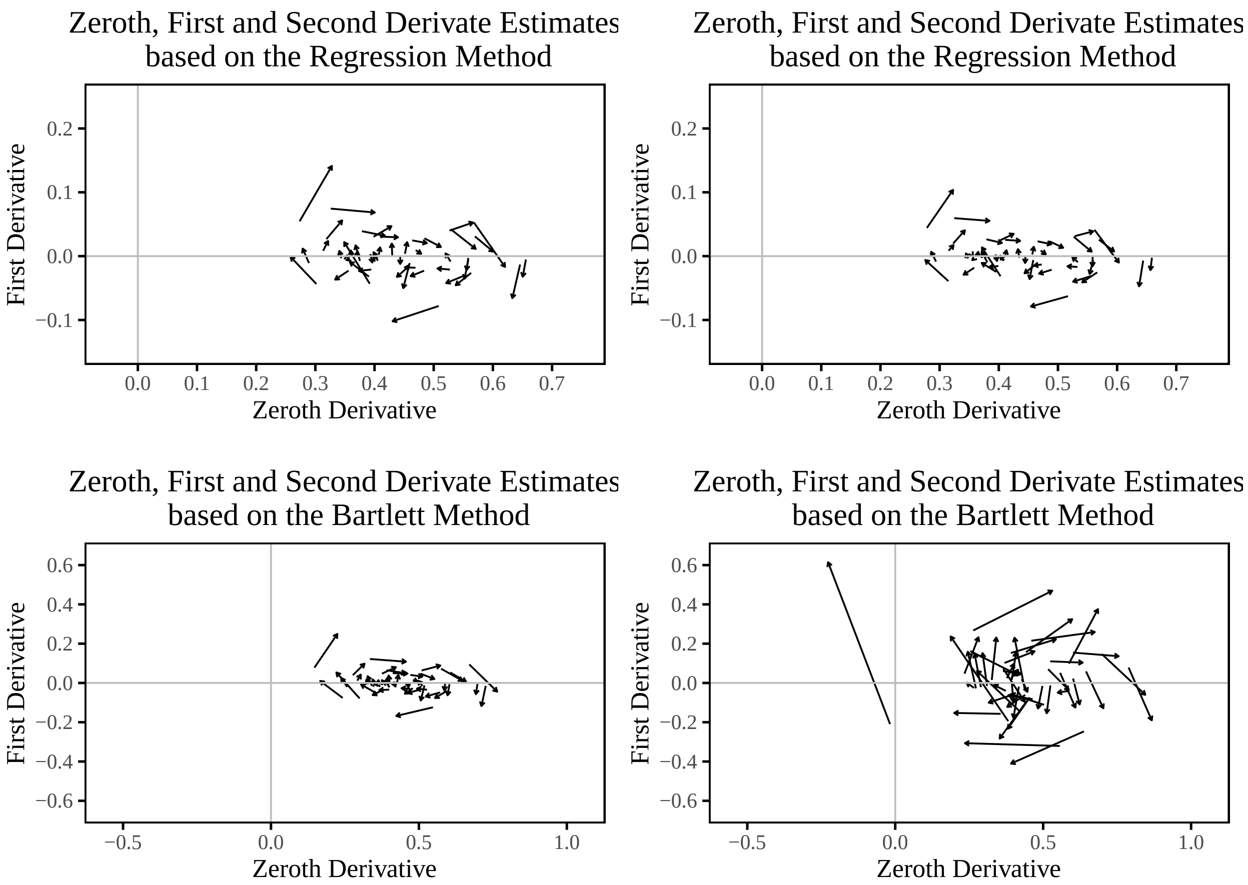

Figure D1. Point estimates for the zeroth, first and second latent derivatives in multivariate SOLDE (left panel) and FOLDE (right panel) models at $D = 6$ based on the regression method (first row) and based on the Bartlett method (second row) for the example case. Note that axes are differently scaled depending on the individual score method.

ics, particularly for the FOLDE model.

Results on the Individual Dynamics for the Example Case Based on the Univariate LDE Modeling

In Figure D2, the dynamics for one exemplary individual are plotted by individual score method and by univariate LDE model. This individual's dynamics may originate from a fix point attractor (the individual's equilibrium), towards which the arrows are spiraling down. The figure highlights the advantage of the regression method over the Bartlett method in visualizing dynamics. For the Bartlett method, dynamics are barely recognizable in the FOLDE model. For the regression method, the pattern of dynamics is the same for the SOLDE and for the FOLDE model.

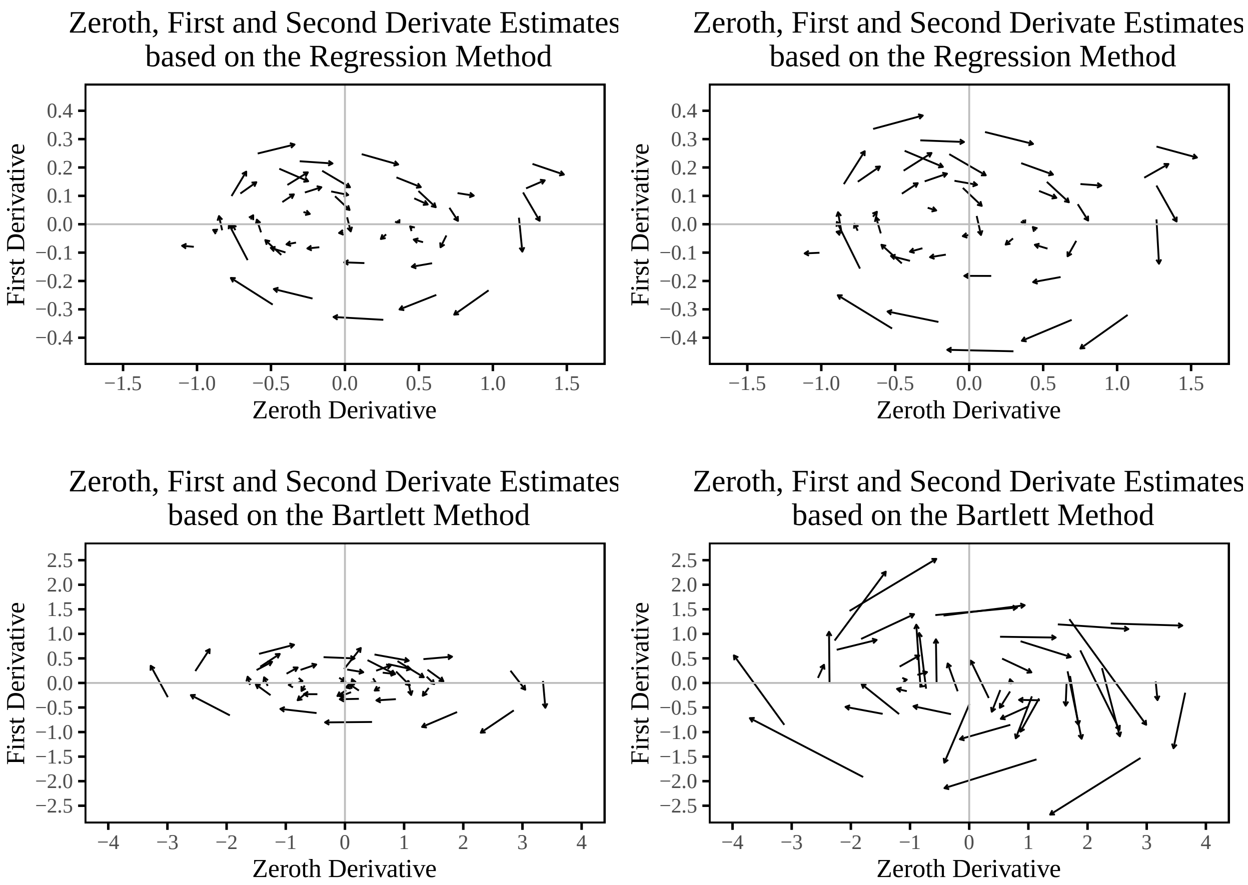

Figure D2. Point estimates for the zeroth, first and second latent derivatives in univariate SOLDE (left panel) and FOLDE (right panel) models at $D = 10$ based on the regression method (first row) and based on the Bartlett method (second row) for the example case. Note that axes are differently scaled depending on the individual score method.

Vector Field Plots for Another Four Individuals

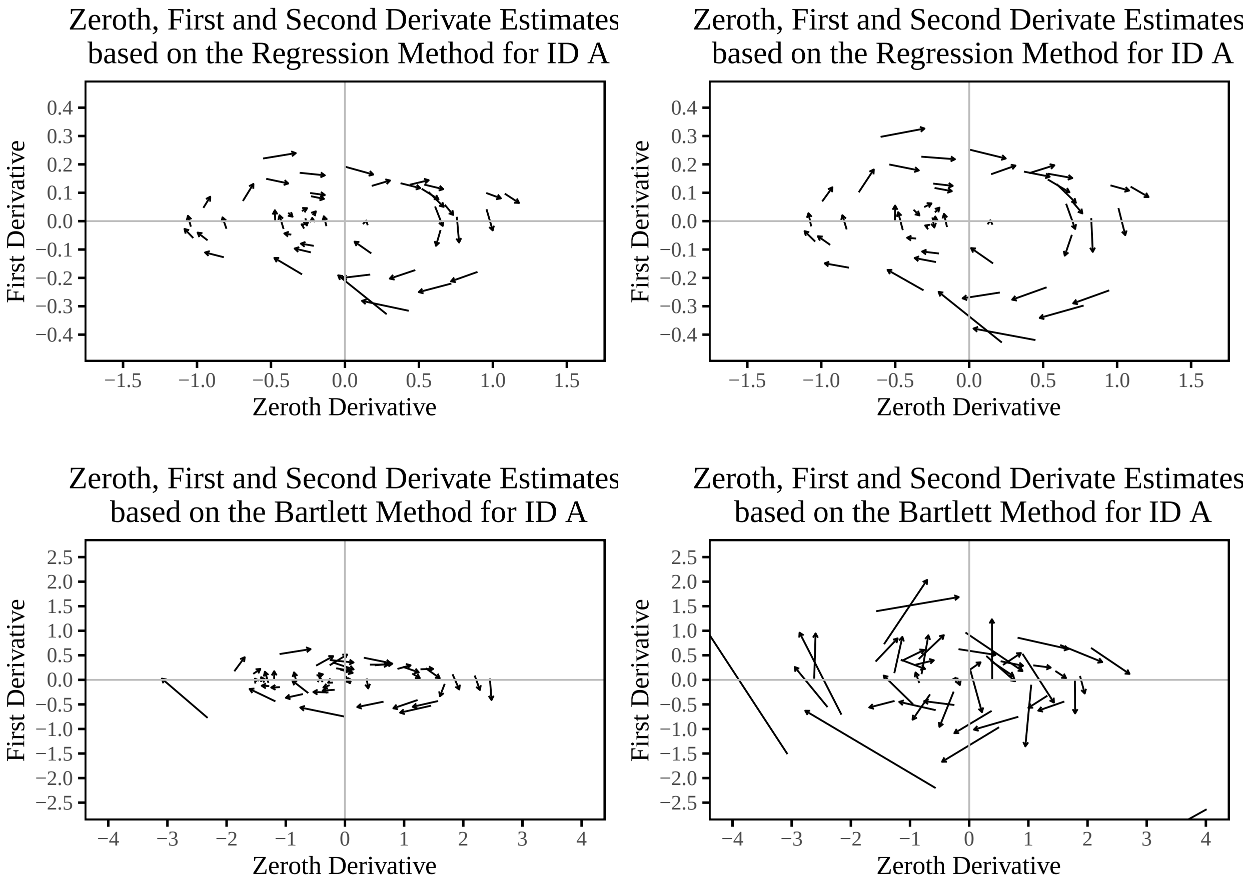

Figure D3. Point estimates for the zeroth, first and second latent derivatives in univariate SOLDE (left panel) and FOLDE (right panel) models at $D = 10$ based on the regression method (first row) and based on the Bartlett method (second row) for individual A. Note that axes are differently scaled depending on the individual score method.

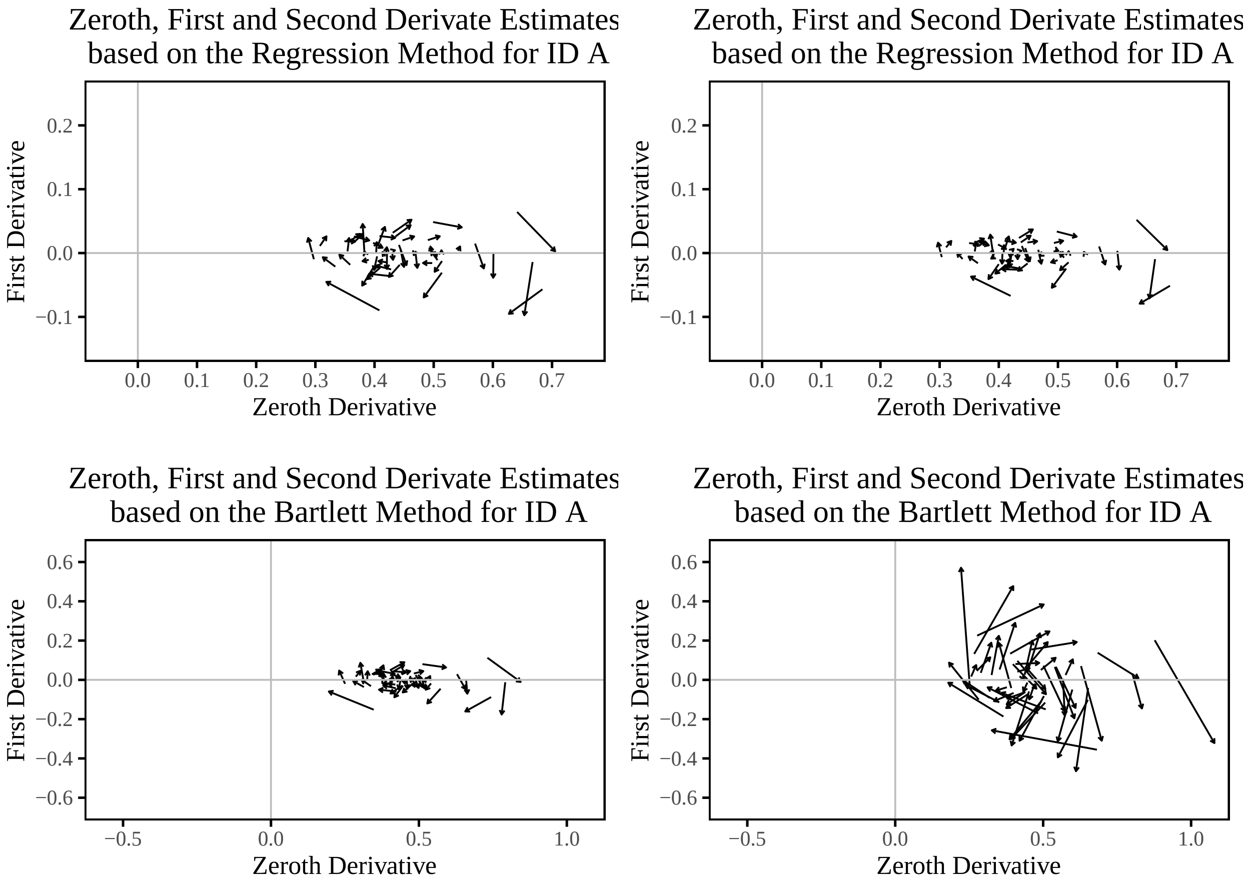

Figure D4. Point estimates for the zeroth, first and second latent derivatives in multivariate SOLDE (left panel) and FOLDE (right panel) models at $D = 6$ based on the regression method (first row) and based on the Bartlett method (second row) for individual A. Note that axes are differently scaled depending on the individual score method.

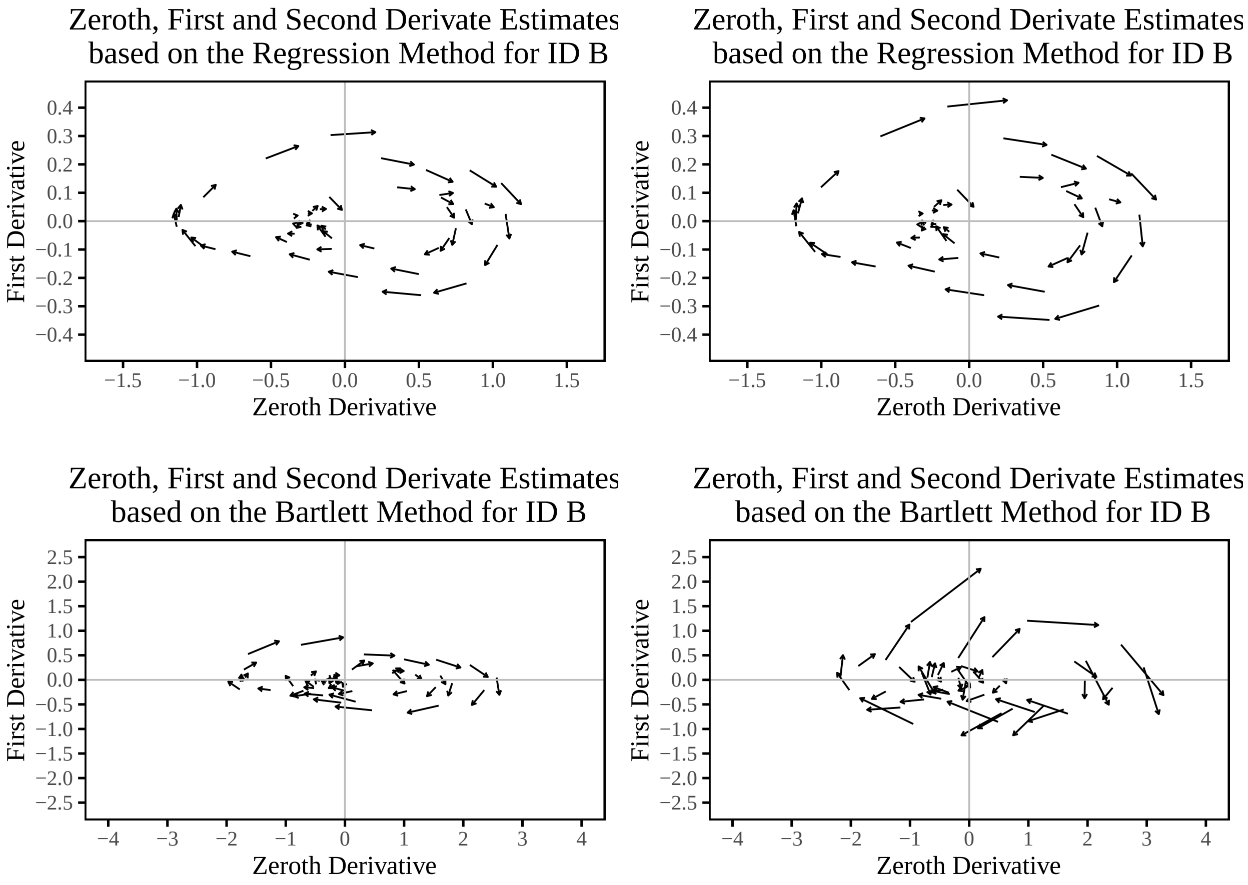

Figure D5. Point estimates for the zeroth, first and second latent derivatives in univariate SOLDE (left panel) and FOLDE (right panel) models at $D = 10$ based on the regression method (first row) and based on the Bartlett method (second row) for individual B. Note that axes are differently scaled depending on the individual score method.

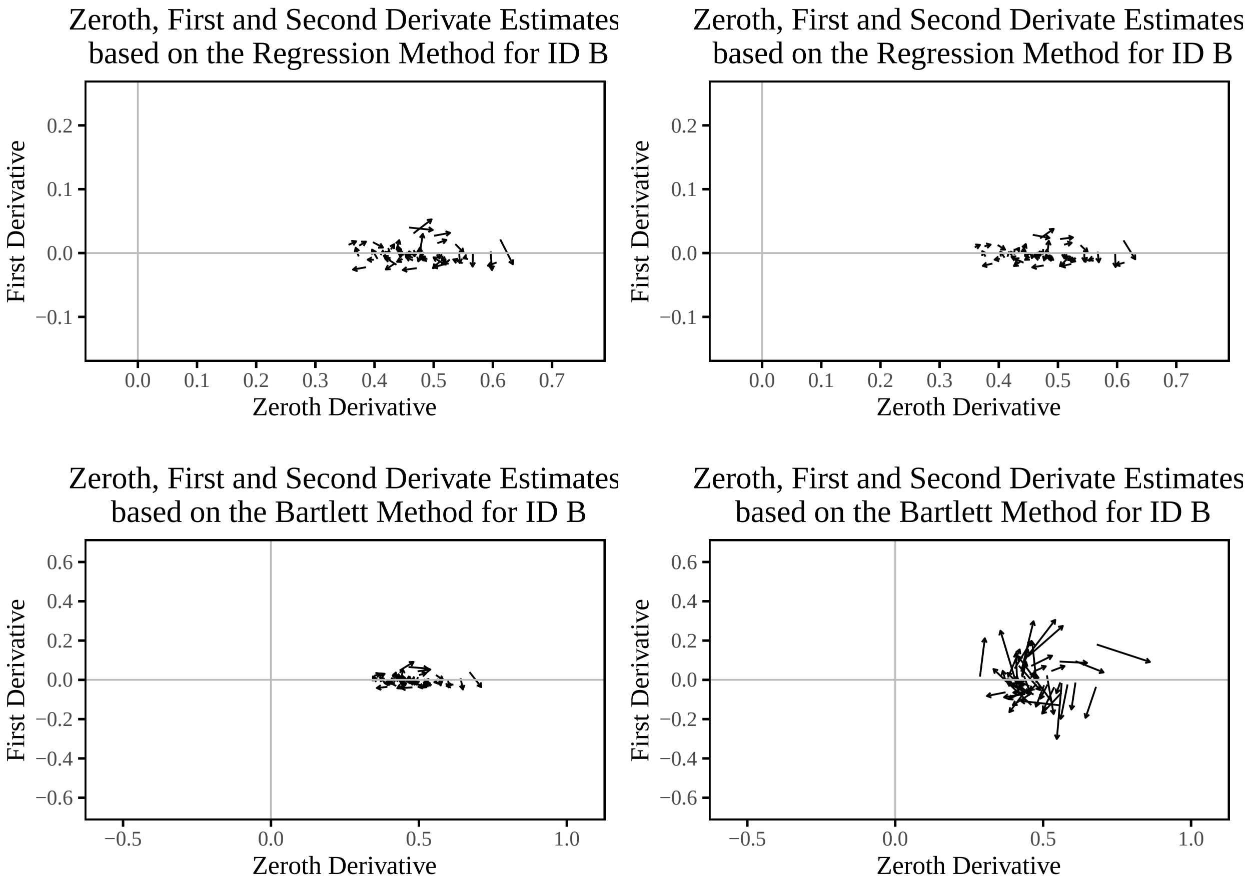

Figure D6. Point estimates for the zeroth, first and second latent derivatives in multivariate SOLDE (left panel) and FOLDE (right panel) models at $D = 6$ based on the regression method (first row) and based on the Bartlett method (second row) for individual B. Note that axes are differently scaled depending on the individual score method.

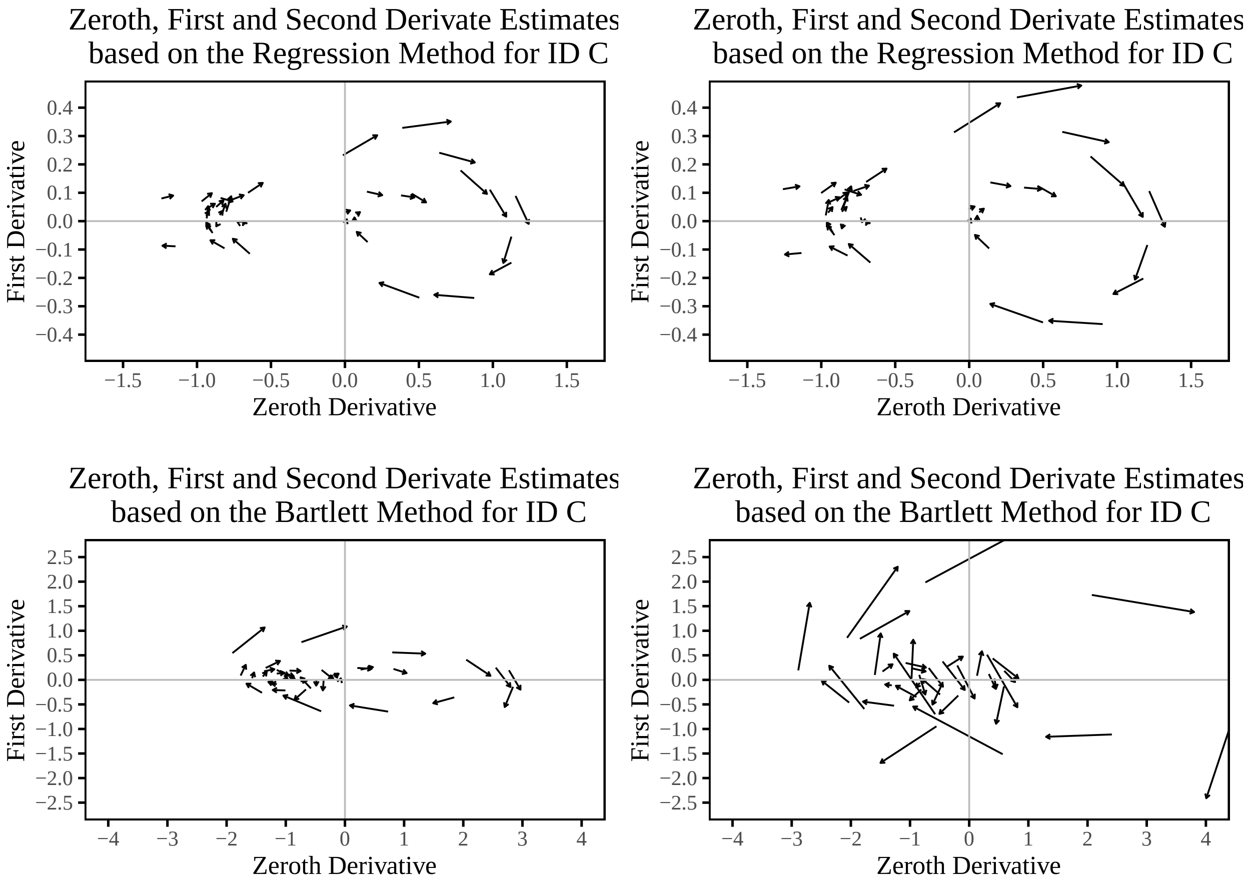

Figure D7. Point estimates for the zeroth, first and second latent derivatives in univariate SOLDE (left panel) and FOLDE (right panel) models at $D = 10$ based on the regression method (first row) and based on the Bartlett method (second row) for individual C. Note that axes are differently scaled depending on the individual score method.

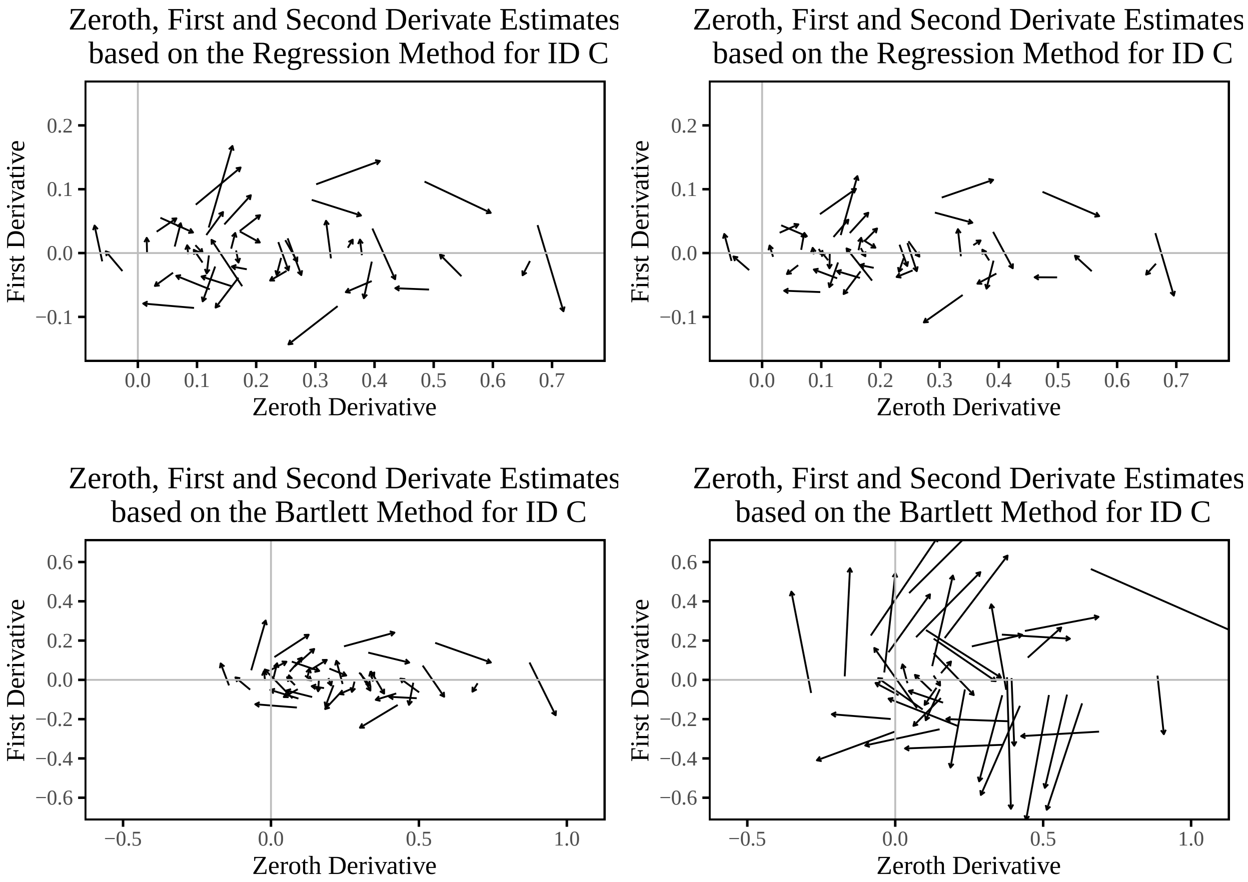

Figure D8. Point estimates for the zeroth, first and second latent derivatives in multivariate SOLDE (left panel) and FOLDE (right panel) models at $D = 6$ based on the regression method (first row) and based on the Bartlett method (second row) for individual C. Note that axes are differently scaled depending on the individual score method.

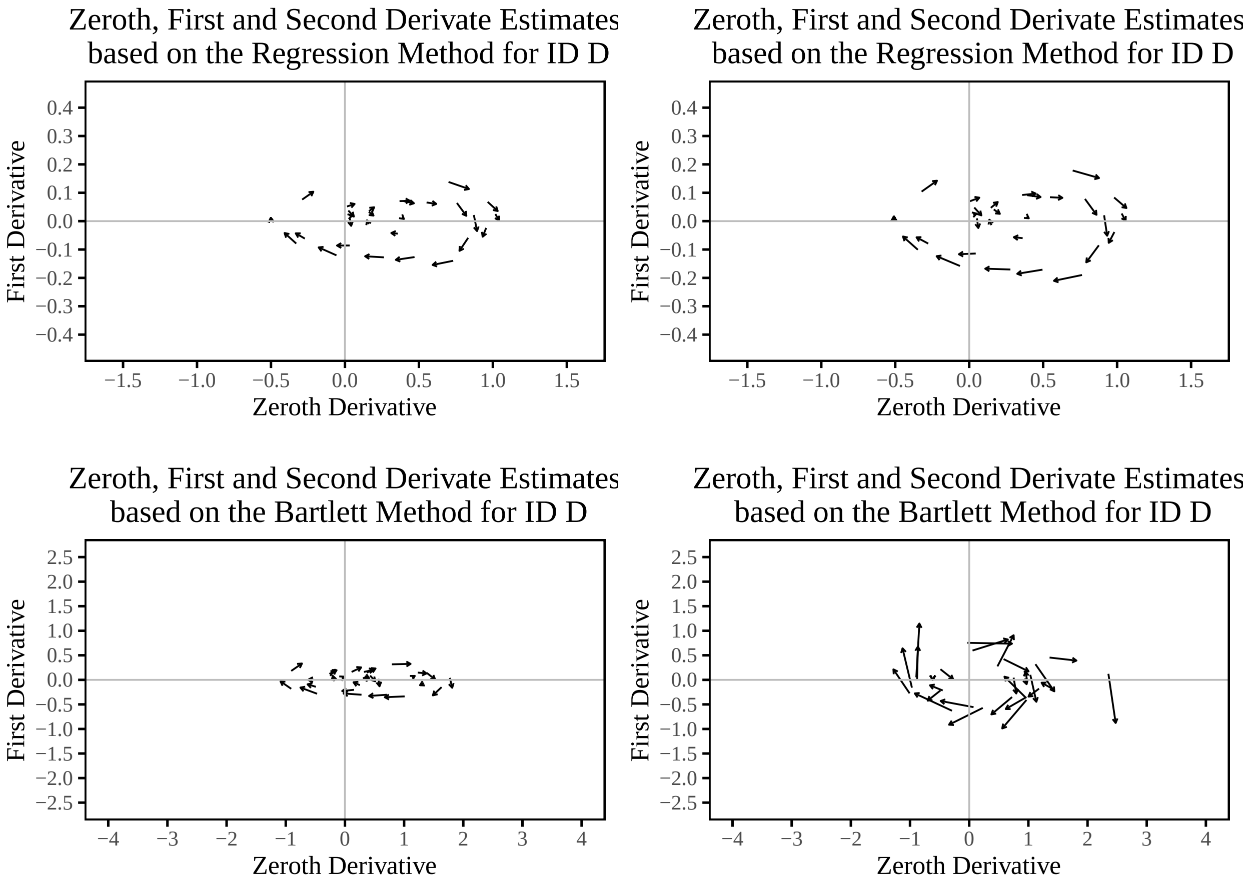

Figure D9. Point estimates for the zeroth, first and second latent derivatives in univariate SOLDE (left panel) and FOLDE (right panel) models at $D = 10$ based on the regression method (first row) and based on the Bartlett method (second row) for individual D. Note that axes are differently scaled depending on the individual score method.

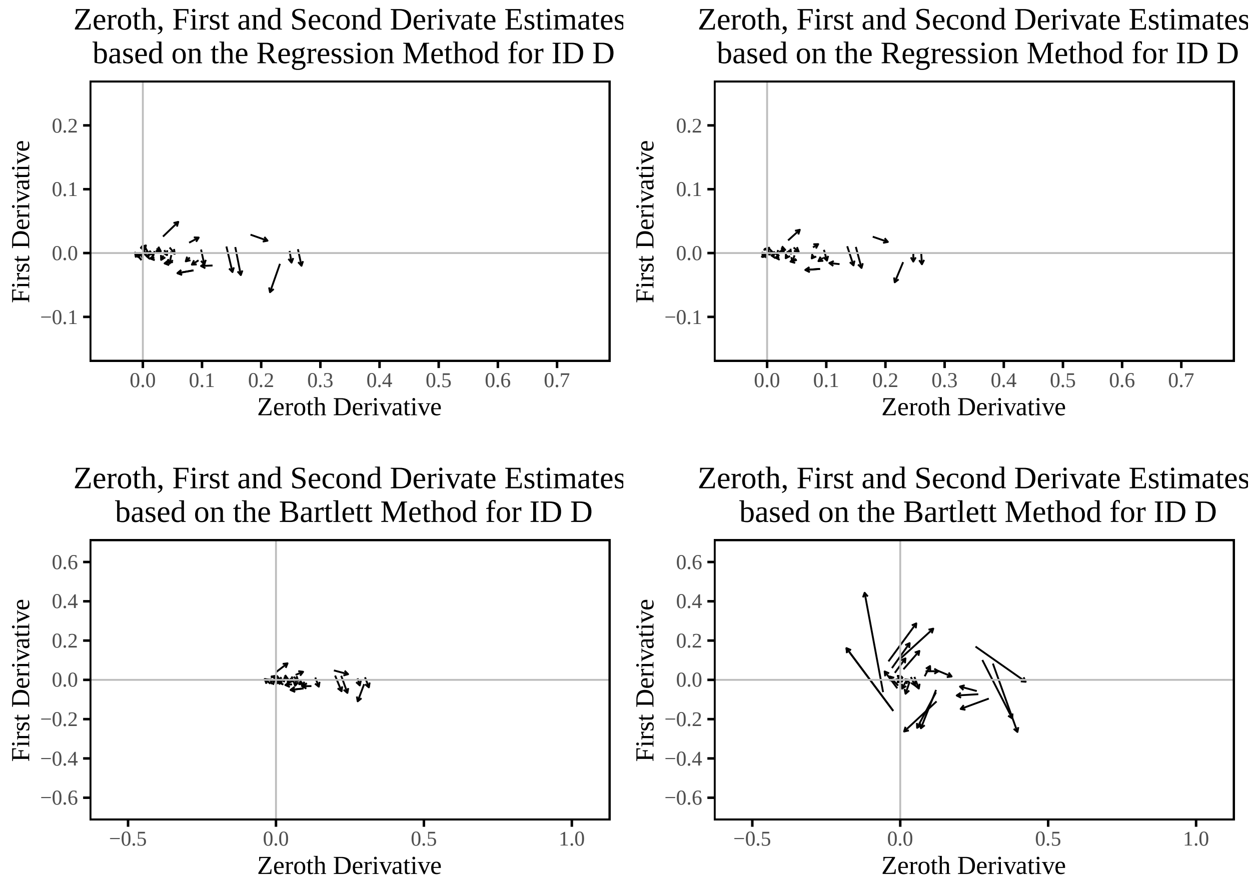

Figure D10. Point estimates for the zeroth, first and second latent derivatives in multivariate SOLDE (left panel) and FOLDE (right panel) models at $D = 6$ based on the regression method (first row) and based on the Bartlett method (second row) for individual D. Note that axes are differently scaled depending on the individual score method.

References

Bartlett, M. S. (1937). The statistical conception of mental factors. *British Journal of Psychology. General Section*, *28*(1), 97–104. doi:10.1111/j.2044-8295.1937.tb00863.x

Thomson, G. H. (1938). Methods of estimating mental factors. *Nature*, *141*(3562), 246–246. doi:10.1038/141246a0

Thurstone, L. L. (1934). The vectors of mind. *Psychological Review*, *41*(1), 1–32. doi:10.1037/h0075959
